# A Polyphasic Approach Reveals Novel Genotypes and Updates the Genetic Structure of the Banana Fusarium Wilt Pathogen

**DOI:** 10.3390/microorganisms10020269

**Published:** 2022-01-25

**Authors:** Diane Mostert, Emmanuel Wicker, Mignon M. de Jager, Saif M. Al Kaabi, Wayne T. O’Neill, Suzy Perry, Chunyu Li, Yi Ganyun, Kenneth G. Pegg, Lizel Mostert, Altus Viljoen

**Affiliations:** 1 Department of Plant Pathology, Stellenbosch University, Stellenbosch 7600, South Africa; 17617030@sun.ac.za (M.M.d.J.); alkaabisaif@hotmail.com (S.M.A.K.); lmost@sun.ac.za (L.M.); altus@sun.ac.za (A.V.); 2 CIRAD, UMR PHIM, 34398 Montpellier, France; emmanuel.wicker@cirad.fr; 3 Plant Health Institute Montpellier, University Montpellier, CIRAD, 34398 Montpellier, France; 4 Ghadafan Agriculture Research Station, Ministry of Agriculture and Fisheries, Seeb 121, Oman; 5 Ecosciences Precinct, Department of Agriculture and Fisheries, Brisbane, QLD 4102, Australia; wayne.oneill@daf.qld.gov.au (W.T.O.); suzy.perry@daff.qld.gov.au (S.P.); susieken@bigpond.net.au (K.G.P.); 6 Institution of Fruit Tree Research, Guangdong Academy of Agricultural Sciences, Guangzhou 510640, China; lichunyu881@163.com (C.L.); yiganjun@vip.163.com (Y.G.)

**Keywords:** banana Fusarium wilt, diversity array technology, phylogeny, *secreted in xylem* genes, vegetative compatibility

## Abstract

*Fusarium oxysporum* f. sp. *cubense* (Foc) is a soil-borne fungus that causes Fusarium wilt, a destructive plant disease that has resulted in devastating economic losses to banana production worldwide. The fungus has a complex evolutionary history and taxonomic repute and consists of three pathogenic races and at least 24 vegetative compatibility groups (VCGs). Surveys conducted in Asia, Africa, the Sultanate of Oman and Mauritius encountered isolates of *F. oxysporum* pathogenic to banana that were not compatible to any of the known Foc VCGs. Genetic relatedness between the undescribed and known Foc VCGs were determined using a multi-gene phylogeny and diversity array technology (DArT) sequencing. The presence of putative effector genes, the *secreted in xylem* (*SIX*) genes, were also determined. Fourteen novel Foc VCGs and 17 single-member VCGs were identified. The multi-gene tree was congruent with the DArT-seq phylogeny and divided the novel VCGs into three clades. Clustering analysis of the DArT-seq data supported the separation of Foc isolates into eight distinct clusters, with the suite of *SIX* genes mostly conserved within these clusters. Results from this study indicates that Foc is more diverse than hitherto assumed.

## 1. Introduction

Fusarium wilt of banana is caused by the soil-inhabiting fungus *Fusarium oxysporum* f. sp. *cubense* (Foc) Smith Snyder and Hansen, regarded as one of the most debilitating plant pathogens in recent history. Foc is a member of the *F. oxysporum* species complex (FOSC), a morphologically related group of fungi that includes both pathogenic and non-pathogenic strains. Pathogenic isolates of *F. oxysporum* are recognised by host specificity and named *formae speciales* (f. spp.) [1]. Within *formae speciales* the pathogen is further divided into races and vegetative compatibility groups (VCGs), based on the ability to cause disease to a particular set of host cultivars and the ability of genetically related individuals to exchange genetic material through heterokaryosis, respectively. The designation of *formae speciales* and races in the FOSC, therefore, is an important attribute to identify and manage pathogenic *F. oxysporum* strains. Foc, for instance, is separated into three different races that affect different banana varieties [2,3] and 24 VCGs [4]. Nineteen of these VCGs are found in Asia, eight in Australia, seven in Africa and seven in the Americas. Because of the greater diversity of Foc in Asia, it is believed that the pathogen evolved with its *Musa* host in this region [5,6,7,8].

In *F. oxysporum*, vegetative compatibility is controlled by at least seven vegetative or heterokaryon incompatibility (*het*/*vic*) loci. Isolates with identical alleles at these *vic* or *het* loci belong to the same VCG [9]. If one or more mutations occur at a *vic* or *het* locus, isolates cannot anastomose and thus belong to different VCGs [10]. VCGs are considered good phenotypic markers to assess diversity in asexually reproducing fungi, but not to measure genetic distance. Still, they are valuable to demonstrate the migration of Foc strains and their introduction into new areas, or to indicate mutations that result in variants of existing VCGs. Some VCGs appear to be confined to certain geographic regions. For instance, VCGs 0121, 0122, 0123, 01217, 01218 and 01219 are found exclusively in Asia [11], VCGs 0129 and 01211 in Australia and Indonesia [12,13], VCG 01210 in Cayman Islands, Cuba and Florida [14] and VCG 01214 in Malawi [15]. Some VCGs are cross-compatible and are referred to as VCG complexes. These include VCG 0120 and 01215, VCG 0124 and 0125, VCG 0129 and 01211, as well as VCG 01213 and 01216 [12].

Several DNA-based techniques have been used to measure genetic distance and study the relatedness among Foc VCGs. These techniques include RFLP analysis [15], RAPD and/or DNA amplification fingerprinting (DAF) analysis [7,16,17], AFLPs [18] and multi-gene sequencing [19,20,21,22]. The phylogenetic analyses of a global collection of Foc isolates indicated that the fungus is polyphyletic and divided into two clades, which represents the two phylogenetic species described for FOSC by Laurence et al. [23]. The two clades are further divided into eight to ten clonal lineages, each consisting of several closely related VCGs [17,20].

DNA-based technologies used to study Foc diversity in the past were limited by a lack of reliability and resolution (RAPD markers) or were labour intensive and not amenable for high-throughput genotyping (AFLP, RFLP). For some, the initial development costs were also very high or required sequence information (SSRs) [24]. Diversity array technology (DArT) [25], combined with next-generation sequencing, provides a unique opportunity to generate thousands of genetic markers with limited genomic resources. DArT markers have been used to study genetic diversity, for linkage mapping, and for population analysis in various crop species (https://www.diversityarrays.com/, accessed on 22 November 2021). It has also been used to investigate the phylogeography of Foc tropical race (TR) 4 [26].

The genome of *F. oxysporum* is compartmentalised and consists of a core genome and an accessory genome [27]. The core genome is mostly conserved among FOSC species, whereas the accessory genome harbours virulence factors and is directly associated with pathogenicity. The horizontal transfer of accessory chromosomes or gene regions is the most accepted hypothesis to explain the polyphyletic origin of host specificity and the emergence of new pathogenic lineages in *F. oxysporum* [27]. The phylogenetic analysis of Foc strains when using genes from the core genes, therefore, does not necessarily infer host-specificity in the banana Fusarium wilt fungus [15,17,19,20,28,29]. The polyphyletic evolution of Foc races, thus, could have occurred through the horizontal transfer of pathogenicity genes such as the *secreted in xylem* (*SIX*) genes. Various *SIX* gene homologues have been correlated with Foc races, while *SIX1* and *SIX9* genes were present in all Foc isolates [21].

Foc isolates that did not fit into known VCGs have been reported before by Mostert et al. [6] and Bentley et al. [17], signifying that the global population of Foc consists of more than the 24 known VCGs. The objectives of this study, therefore, were to (1) determine the pathogenicity of *F. oxysporum* strains collected from banana that do not fit known Foc VCGs; (2) describe their VCG identities; (3) determine their *SIX* gene homologue profile; and (4) compare them with known Foc VCGs using housekeeping gene sequences and DArT sequencing.

## 2. Materials and Methods

### 2.1. Fungal Isolates

A total of 169 isolates, collected from banana pseudostems, were investigated in this study (Table S1). These included isolates previously identified as representatives of the 24 known Foc VCGs [6,20]; isolates that were previously described as incompatible with known VCGs [6,17,30,31]; isolates newly collected in the Philippines, the Sultanate of Oman and Mauritius that did not match known Foc VCGs; and six *F. oxysporum* endophytes of which two were collected in Tanzania by Karangwa et al. [32] and four in Australia. The Australian endophytes were PCR-amplified with Foc TR4-specific PCR primers [33]. All the isolates, except for the endophytes from Australia, are maintained in the Fusarium culture collection (CAV) at the Department of Plant Pathology, Stellenbosch University in South Africa.

### 2.2. Cultural, Morphological and Molecular Identification

The isolates were grown on carnation leaf agar (CLA), synthetic low-nutrient agar (SNA) and potato dextrose agar (PDA) to induce sporulation under continuous cool-white illumination (Osram L18W/840) for 7 d at 25 °C. Morphological features produced on CLA such as the presence and abundance of micro- and macroconidia, chlamydospores and coiled hyphae were examined using light microscopy [1,32].

### 2.3. Pathogenicity Testing

One hundred and one *F. oxysporum* isolates that were collected from banana were tested for pathogenicity to banana seedlings according to the methodology described by Viljoen et al. [34] (Table S1). Millet seeds were washed and soaked in tap water for 6 h, the water was drained and the millet seeds autoclaved in 415 × 600 mm autoclavable bags (LabFriend Pty Ltd, Sydney, Australia) on two consecutive days for 20 min at 120 °C. Twenty to thirty mycelial plugs (10 × 10 mm) from the cultures, grown on PDA for 4–5 d at 25 °C, were then used to inoculate 500 g sterile millet seeds in 1 L Schott bottles. The bottles were incubated in the dark at 25 °C for 10–14 d and shaken every third day to ensure that the fungus properly colonised the millet seeds. Three 10 cm tissue culture-derived Cavendish and Gros Michel plants were used for pathogenicity testing. Plants were hardened off in a greenhouse before inoculation for 2 months. When plants were ready, they were uprooted and replanted in a 1.4 L pots containing a steam sterilised soil mixture (1 potting soil: 1 peat: 1 sand) with 10 g colonised millet kg^−1^ of soil. The positive control used in the experiment was a pathogenic Foc isolate (VCG 0120; CAV 179) and the negative control was sterile millet seed. After inoculation, the plantlets were kept in the greenhouse for 6 weeks at 25 °C. Isolates were considered pathogenic if they caused discolouration of the inner rhizome. The inoculated fungus was then re-isolated from the edges of the discoloured tissue and morphological identification conducted to complete Koch’s postulates. The experiment was repeated.

### 2.4. Nit-Mutant Generation and VCG Testing

VCG testing was performed as described by Leslie and Summerell [1]. Nitrate non-utilizing (*nit*) mutants were first generated by transferring Foc isolates grown on PDA to minimal media (MM) amended with 1.5–4.5% KClO_3_ [35]. The isolates were then incubated for 7–21 d at 25 °C, and KClO_3_-resistant sectors that developed were transferred to MM slants. Cultures that grew sparsely were classified as *nit*-mutants and were further characterised on selective media containing one of four different sources of nitrogen [9].

The VCG identities of the unknown Foc isolates were determined by pairing their *nit-1* and/or *nit-3* mutants on MM with Nit-M testers of the 24 known VCGs, and vice versa [20]. The tester strains of the known Foc VCGs were obtained from Dr. Suzy Perry and Mr. Wayne O’Neill (Queensland Department of Agriculture and Fisheries, Brisbane, QLD, Australia), Prof. Randy Ploetz (University of Florida, Homestead, FL, USA) and Dr. Kerry O’Donnell (United States Department of Agriculture, Peoria, IL, USA). Pairings were also made between *nit-1* or *nit-3* mutants of the unknown isolates and their own Nit-M mutants to investigate vegetative self-incompatibility [1], as well as with Nit-M mutants of other unknown isolates to identify new VCGs [10]. Isolates that formed heterokaryons between *nit-1* or *nit-3* and Nit-M mutants were considered members of the same VCG. Isolates that were self-compatible in *nit1*/Nit-M complementation tests, but did not pair with any other VCG, were identified as single-member VCGs (SMVs). All the mutants generated for Foc isolates are also maintained on MM slants in the culture collection of the Department of Plant Pathology, Stellenbosch University, while standard tester sets for distinct VCGs and SMVs are maintained in 15% glycerol at −80 °C.

### 2.5. Multi-Gene Phylogeny

Isolates of *F. oxysporum* were grown on PDA for 1 to 2 weeks, whereafter mycelia was harvested. DNA was then extracted from the mycelia using the Wizard SV Genomic DNA Purification System Kit (Promega, Madison, IL, USA), and the DNA concentration quantified using a Nanodrop spectrophotometer (NanoDrop, Wilmington, NC, USA). The DNA was thereafter stored in Eppendorf tubes at −20 °C until use.

Three gene regions of each isolate were partially sequenced including the RNA po-lymerase largest subunit gene (*rpb1*) using primers RPB1-Fa and RPB1-G2R [36], the RNA polymerase second largest subunit gene (*rpb2*) using primers RPB2-5f2 and RPB2-7cr [36], and the translation elongation factor 1-alpha gene (*tef1*) using primers EF1 and EF2 [19]. The PCR products were purified using the High Pure PCR Product Purification Kit (Roche Applied Biochemicals, Indianapolis, IN, USA), and sequenced in both directions with the original PCR primers. Raw sequences were manually checked and edited where necessary with Geneious version 5.04 (Biomatters LTD, Auckland, New Zealand). Representative sequences of unknown Foc VCGs and SMVs were uploaded to GenBank (Table S1).

Multiple sequence alignments were constructed using MAFFT v5.85 (http://align.bmr.kyushu-u.ac.jp/mafft/online/server/, accessed on 8 September 2021), with the L-INS-i option effective [37,38]. Datasets consisted of combined sequences of the RPB1, RPB2 and TEF gene regions of representative isolates of the unknown VCGs identified in this study and the study of Bentley et al. [17]. Representatives of the 24 known Foc VCGs [20,22] and one isolate representing each of FOSC Clades 1–5 described by O’Donnell et al. [39,40] were included as reference strains. *Fusarium fujikuroi* (NRRL 13566) was downloaded from GenBank and included as outgroup.

The data of three genes that were sequenced were concatenated in Geneious Prime v2019.2.3, with the phylogenetic inference based on maximum parsimony (MP) and maximum likelihood (ML). ML analysis was performed using the PhyML v3 [41] available at the Montpellier bioinformatics online platform (http://www.atgc-montpellier.fr/phyml/, accessed on 8 September 2021), and the best fit evolutionary model selected with the Smart model selection tool in PhyML [42] with the Akaike Information Criterion setting. The general time-reversible model, with correction for invariable sites and gamma correction for among-site variation (GTR + I + G), was selected for the data. Maximum parsimony analysis was performed with the heuristic search option effective in PAUP v4 [43], using only parsimony informative sites [44,45]. Bootstrap confidence values for both the MP and ML analysis were based on 1000 replicates. Trees were visualised and edited with Adobe Acrobat Pro D and FigTree v1.4.4 (http://tree.bio.ed.ac.uk/software/figtree/, accessed on 8 September 2021) software.

### 2.6. Genome-Wide Genotyping

#### 2.6.1. DArT Sequencing

DNA of 87 *F. oxysporum* isolates were sent for sequencing on the Diversity Array Technology platform in Canberra, Australia. The set included representatives of the 24 known Foc VCGs and the newly identified Foc VCGs and SMVs reported in this study (Table S1). The DNA of six non-pathogenic *F. oxysporum* isolates from banana collected by Karangwa et al. [31] and Accessions BRIP 62577, 62618, 62833 and 62834 from the Queensland Plant Pathology Herbarium Accessions were also included.

The data were analysed using DArTsoft v.7.4.7 (DArT P/L, Canberra, Australia). The DArTseq and SNPs markers were scored using DArTsoft as binary data (1/0), indicating the presence or absence of a marker in the genomic representation of each sample. The DArT software automatically computes quality parameters for each DArTseq and SNP marker, such as call rate, polymorphic information content (PIC) and reproducibility. Based on these parameters, the DArTseq and SNP data were further analysed and filtered using the *dartR* package [46]. First, monomorphic loci were removed from both data sets, and only individual genomes with a repeatability index above 0.99 were kept. Both the remaining loci and individual genomes were kept for downstream analyses when their call rates were higher than 0.9 and 0.75, respectively [47,48]. To calculate the correlation between DArT and SNP analysis, a distance-based matrix was created between each of the genotypes using the Jaccard distance function in R software (The R Development Core Team, 2013). The Jaccard distance-based distance matrices were used to correlate DArT and SNP data sets using the CADM function of the *ape* package. The significance of this correlation was statistically assessed with 9999 bootstrap permutations. From this initial quality screening, 83 genomes were retained for downstream analyses.

#### 2.6.2. Phylogenetic Analysis Based on DArT-Seq Data

Phylogenetic analysis was performed on the SNP data only, and the SNP data transformed using the gl2fasta function in *dartR* package. The dataset was then used to generate an ML phylogeny using RAxML 8.2.11, with the GTR + CAT substitution model, in Geneious prime version 2019.2.1. The robustness of the nodes was tested using 100 bootstrap permutations.

A phylogenetic network was built using the software SplitsTree 4.15.1 [49]. The multifastA file was first converted to a nexus file (haploid autosomal DNA) using dnaSP6 [50]. The phylogenetic network was then generated using the Neighbor-Net algorithm. To determine the probability of recombination, a pairwise homoplasy index (PHI) test, Φ_w_ [51], was performed and implemented in Splitstree4 [49].

#### 2.6.3. Genetic Structure Analysis

To determine the genetic relationship among Foc isolates a fast maximum likelihood method, *snapclust*, was used. *Snapclust* allies the advantages of both model-based and geometric approaches [52]. The DArTseq and SNP data were first converted into a genind object using the *dartR* package [46]. The optimal number of clusters (k) were estimated using both the Akaike, Kullback and Bayesian Information Criterion with the *adegenet* package version 2.1.1 [53] and the snapclust.choose.k function [52]. Ten runs of the expectation-maximisation (EM) algorithm were then used to estimate the probability of assignment (Q) of each individual into each of the k inferred clusters. All isolates were assigned to one single *snapclust* cluster (SC) with good confidence (Q value above 0.99).

To describe the clusters identified, a discriminant analysis of principal components (DAPC) was performed that assigned the SC as priori groups [52]. DAPC does not assume a population genetics model but attempts to summarise genetic differentiation between groups identified by *snapclust*. It then transforms the data using PCA and performs discriminant analysis on the number of principal components retained. The study determined the optimal number of principal components (PC) by using the cross-validation with the *adegenet* package. Different PC numbers were tried, and the quality of the corresponding DAPC was assessed by cross-validation. The number of PCs associated with the lowest mean squared error (between 20 replicates) was then retained in the DAPC.

### 2.7. Presence of SIX Gene Homologues in Foc

Putative effector genes in the known and unknown Foc VCGs were identified by verifying the presence or absence of *SIX*-gene homologues with PCR. The *SIX*-gene homologue data for the known Foc VCGs reported by Czislowski et al. [21] were included for analysis.

## 3. Results

### 3.1. Cultural and Morphological Identification

The *F. oxysporum* isolates in this study produced microconidia in false heads on short monophialides that were typically single-celled and kidney-shaped. Their most distinctive characteristic was the production of single or pairs of chlamydospores with coarse protective walls. The *F. oxysporum* isolates displayed a cream to dark purple colony colour on PDA when viewed from the bottom of Petri dishes.

### 3.2. Pathogenicity Testing

All the isolates tested caused disease symptoms characteristic of Fusarium wilt in Cavendish and/or Gros Michel banana plants within 6 weeks. This included leaf yellowing and a reddish-brown discolouration of the inner rhizome. Plantlets inoculated with sterile millet seed did not develop any symptoms. The isolates were successfully re-isolated from diseased rhizomes and confirmed as *F. oxysporum*. The ability of the *F. oxysporum* isolates to cause disease to banana confirmed their status as Foc strains.

### 3.3. VCG Testing

Three of the 13 Foc genotypes described by Bentley et al. [17] were known Foc VCGs. Genotype 4 (collected in Malaysia) belonged to VCG 01222, Genotype 13 (collected in Vietnam) belonged to VCG 01221, and Genotype 14 (collected in Vietnam) fit into the VCG complex 0128/01220 (Table S1). When Bentley et al. [17] described these genotypes, Foc VCGs 01221-01224 were not yet named. Four of the genotypes were SMVs and were designated SMV-1 (Genotype 2), SMV-2 (Genotype 3), SMV-3 (Genotype 7) and SMV-4 (Genotype 10). These SMVs were self-compatible, but not compatible to any of the known or new VCGs described in this study. The remaining six genotypes were compatible to multiple isolates collected in Asia and the Middle East. These genotypes were designated VCG 01225 (Genotype 5) that included three isolates, VCG 01226 (Genotype 6) that included six isolates, VCG 01227 (Genotype 8) that included 21 isolates, VCG 01228 (Genotype 9) that included 13 isolates, VCG 01229 (Genotype 11) that included three isolates, and VCG 01230 (Genotype 12) that included two isolates. VCGs 01227 and 01228 appeared to be widespread in the Philippines.

Several new Foc VCGs were also described. These were VCG 01231 with 12 isolates collected in the Philippines, VCG 01232 with two isolates collected in India, VCG 01233 with one isolate from India, two from Sri-Lanka and 19 from Oman, VCG 01234 that included three isolates from Sri-Lanka, VCG 01235 with two isolates from Malaysia, VCG 01236 with two isolates from Bangladesh, VCG 01237 with 10 isolates from the Philippines, and VCG 01238 with seven isolates from the Philippines (Table S1). Thirteen Foc isolates were identified as single member VCGs, and designated Foc SMVs 5–17 (Table S1).

### 3.4. Multi-Gene Phylogenetic Analysis

**Figure 1:**
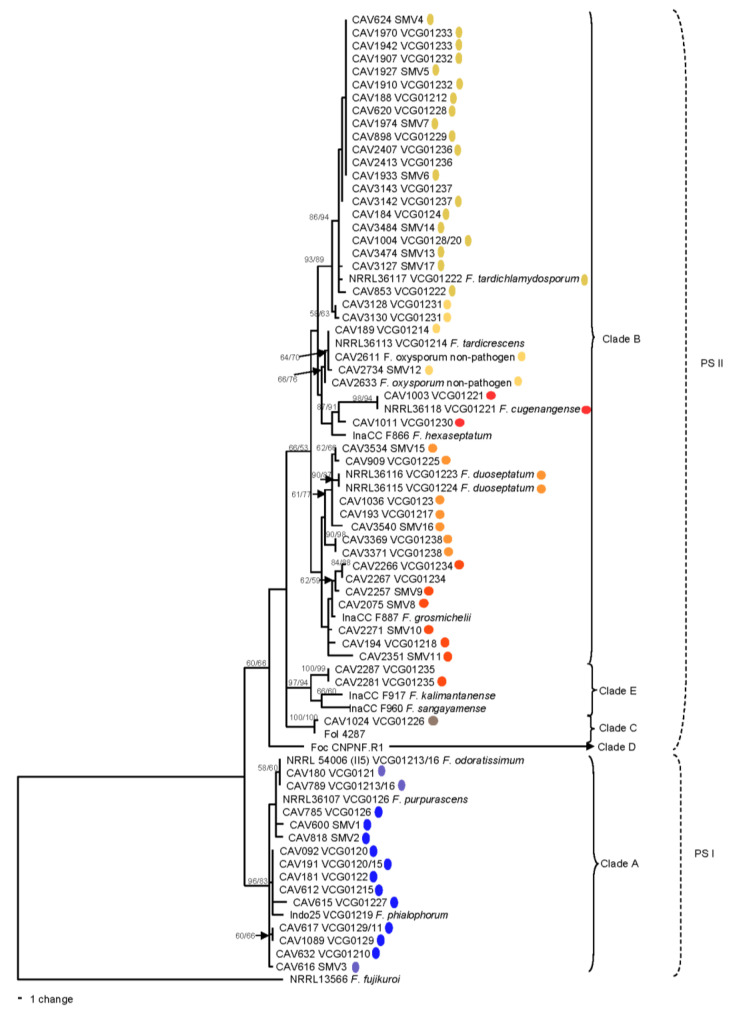
Maximum parsimony tree inferred from the combined data sets of the *rpb1*, *rpb2* and *tef1* gene regions for *Fusarium oxysporum* f. sp. *cubense* isolates. Bootstrap values generated from the maximum parsimony, followed by maximum likelihood analysis (>60%), are shown at the internodes. Clade designation (A–E) is according to O’Donnell et al. [40], while the phylogenetic species boundary sensu Laurence et al. [23] is indicated (PS I and PS II). Culture collection number, Vegetative compatibility group (VCG) or single member VCG, are indicated on tips. Snapclust (SC) grouping is indicated in different colours. The tree is rooted with *F. fujukuroi* NRRL 13566.

The combined phylogeny consisting of *tef1*, *rpb1* and *rpb2* gene sequences separated Foc isolates into four of the FOSC clades described by O’Donnell et al. [40], namely Clades A, B, C and E (Figure 1) and the two phylogenetic species described by Laurence et al. [23], namely PSI and PSII (Figure 1). In Clade A, SMV1 and SMV2 grouped with VCGs 0121, 01213/16 and 0126. VCG 01227 and SMV3 formed part of the VCGs 0120/15, 0122, 01210 and 01219 lineage. Two VCG 0129/11 isolates, however, formed part of a separate lineage with low bootstrap support (Figure 1).

The majority of the new Foc VCGs and SMVs formed part of Clade B. VCGs 01228, 01229, 01232, 01233 and 01236–01238, and SMV5, 6, 7 and 13, all grouped with VCGs 0124, 0125, 0128, 01212, 01220 and 01222 in the same lineage and good bootstrap support (MP = 86%/ML = 94%). VCG 01231 grouped basal to this lineage with bootstrap support (MP = 93%/ML = 89%). VCG 01225 and SMV 15 formed one subgroup, and VCGs 0123, VCG 01217, 01223, 01224 and SMV 16 the other subgroup of the same lineage. SMV 12 grouped with VCG 01214 and two non-pathogenic *F. oxysporum* isolates from Tanzania. VCG 01221 formed an independent lineage with VCG 01230 as a basal group, with good bootstrap support (MP = 89/ML = 91%). VCGs 01234 and 01238, as well as SMV 9, grouped in well-supported independent lineages. SMVs 8, 10 and 11, and VCG 01218 were not well resolved within Clade B.

Two VCGs grouped outside FOSC Clades A and B. VCG 01235 formed part of FOSC Clade E, and VCG 01226 was included in FOSC Clade C where it grouped with *F. oxysporum* f. sp. *lycopersici* isolate Fol 4287 with good bootstrap support (MP = 100%/ML = 100%).

### 3.5. DArT Sequencing

A total of 77,267 DArT loci and 26,399 SNP polymorphic markers were generated by DArT sequencing. The call rate for the 87 Foc genomes ranged from 0.73–1.0 with an average of 0.985 for DArT (Figure S1A), and between 0.54 and 1.0 with an average of 0.962 for SNPs (Figure S1B). After filtering, the genomes of CAV 2266, CAV 2287, CAV 2413 and CAV 3143 were removed, and the dataset consisted of 75,788 binary DArT loci and 5196 binary SNPs from 83 Foc genomes. The DArT and SNP distance matrices were highly correlated, with a Mantel correlation coefficient of 0.823 (*p* = 0.0001), indicating significant congruency between the two datasets (Figure S2).

### 3.6. Genome-Wide Genotyping

**Figure 2:**
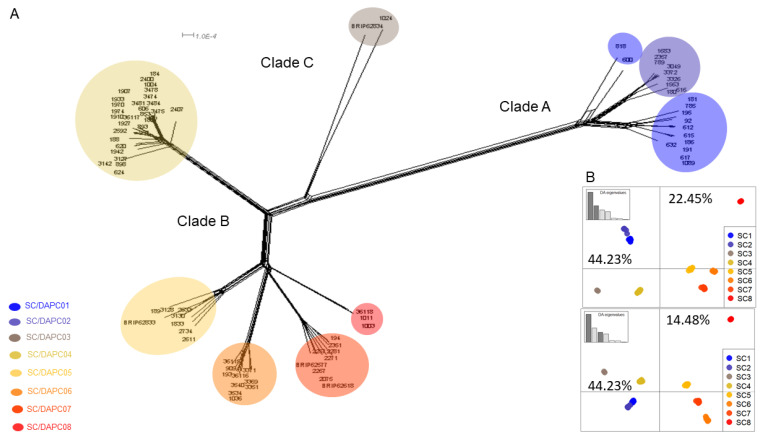
Clustering analysis indicating *Fusarium oxysporum* f. sp. *cubense* (Foc) consists of eight distinct genetic clusters. (**A**) SplitsTree clustering network based on the distance between 83 Foc isolates as indicated by 5240 binary SNP markers. Different snapclust (SC) or discriminant analysis of principal component (DAPC) groups are shown by different colours, while individual isolates are indicated on each root tip. Foc clade identities as determined by O’Donnell et al. [40] are indicated on branches. (**B**) tion of the eight genetic on the plan DA1-DA2 (**top**) and DA1-DA3 (**bottom**). The percentages on each scatterplot indicate the inertia rate of each axis (which can be interpreted as the percentage of variation explained by the axis), while the SC groups are shown by different colours, and dots represent individual genotypes.

Snapclust analysis divided the 83 Foc isolates into eight clusters (Figure 2A and Figure S3). According to the matrix of group membership probabilities (Q), each individual belonged to a single cluster (Q above 0.99). DAPC further summarised the genetic differentiation between the SCs (Figure 2B). SC1 and SC2 clustered together on the negative side of the horizontal axis 1. CAV 600 (SMV1) and CAV618 (SMV2) grouped basally to SC1 and SC2 and presented reticulate phylogenies. SC4 grouped distant from SC5-8 both on the NeighborNet network (Figure 2A) and the SC/DAPC scatterplots (Figure 2B). SC5, SC6 and SC7 grouped together on the positive part of axis 1, while SC8 was distant from the others in the top-right corner of both DAPC scatterplots (Figure 2B).

The correspondence of each SC group to Foc VCGs and SMVs is summarised in Table 1. The pairwise homoplasy index (PHI) test performed on the Foc SNP dataset gave a mean Φ_w_ = 0.1597, which provided statistical evidence for recombination (*p*-value = 0.0154). The PHI test was then applied to each of the SC clusters except SC3 and SC8, which contained only two and three genomes, respectively. From this test, signatures of recombination were detected in SC6 only (Table S2), while reticulate phylogenies were also observed in SC4-SC7 (Figure S4a–i).

### 3.7. Phylogenetic Analysis of SNP Data

**Figure 3:**
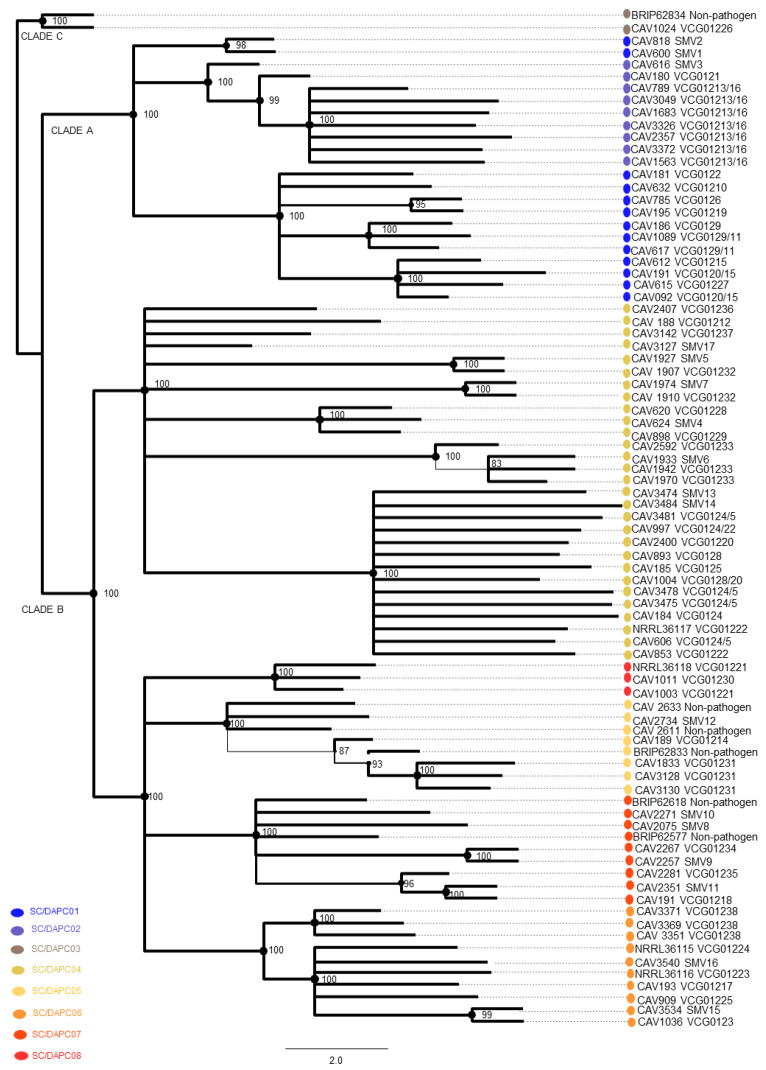
Maximum likelihood analysis of 83 *Fusarium oxysporum* f. sp. *cubense* isolates based on single nucleotide polymorphisms. Bootstrap values generated from the maximum likelihood analysis are shown at the internodes. Clade designation (A–C) according to O’Donnell et al. [40] and vegetative compatibility group (VCG) or single member VCG are indicated on nodes.

All Foc VCGs, including the newly described ones, grouped into eight monophyletic lineages according to SNP data ML phylogeny (Figure 3). Isolates in VCG 01213/16, VCG 01231 and VCG 01238 produced near-clonal clusters in SC2, SC5 and SC6, respectively. If not in near-clonal clusters, isolates in the same VCGs were grouped in coherent branches, such as VCG 0129/11 and VCG 0120/15 in SC1, VCG 01233 in SC4, and VCG 01221 in SC8. VCGs 0124/5, 0128, 01220 and 01222, and SMVs 13 and 14, formed a comb-shaped sub-lineage in SC4. Several VCGs could not be resolved in SC4 and were polytomic, including VCGs 01212, 01236 and 01237, and SMV17. There was contrast in VCG diversity between Foc clades, with the highest diversity found in Clade B (SC 4 to 8). Twelve of the 20 newly described VCGs grouped in SC4, while most of the VCGs in SC5, 6, and 7 were new. 

### 3.8. Correspondence between Multi-Gene and DArT-Seq SNP Phylogenies

The grouping of Foc VCGs and SMVs was mostly consistent between the multi-gene (Figure 1) and SNP (Figure 3) phylogenies. The SC1 lineage included Foc strains commonly referred to as Foc subtropical race (STR) 4, such as VCGs 0120/15, 0122, 0126, 0129/11, 01210 and 01219 (Figure 3). The new VCGs and SMVs described in SC1 were VCG 01227 and SMVs 1 and 2. In the SNP phylogeny, VCG 0121 and SMV 3 clustered with VCG 01213/16, which is commonly referred to as Foc TR4, in SC2 (Figure 3). In the multi-gene phylogeny, however, SMV 3 grouped with the Foc STR4 VCGs 0120/15, 0122 and 01219 in SC1 (Figure 1). The Clade C VCG 01226 clustered with a non-pathogenic isolate, BRIP62834, in SC3. The SC4 cluster, which commonly includes Foc races 1 and 2 isolates, included the newly described VCGs 01228, 01229, 01232, 01233, 01236 and 01237, and the SMVs 4, 5, 6, 7, 13, 14 and 17, which clustered with the known VCGs 0124, 0125, 0128, 01212, 01220 and 01222 (Figure 1 and Figure 3). In the SNP phylogeny, VCG 01231 and SMV 12 clustered with three non-pathogenic *F. oxysporum* isolates and VCG 01214 in SC5 (Figure 3). In the multi-gene phylogeny however, VCG 01231 clustered basal to the SC4 VCGs (Figure 1). SC6 grouped four newly described VCGs 01225 and 01238 and SMVs 15 and 16 with the known VCGs 0123, 01217, 01223 and 01224. SC7 comprised of VCGs 01234 and 01235, and SMVs 8, 9, 10 and 11, which clustered with two non-pathogenic *F. oxysporum* isolates and VCG 01218 in the SNP phylogeny (Figure 3). In the multi-gene phylogeny however, VCG 01235 clustered separate from other Foc isolates in FOSC Clade E. SC8 comprised of VCG 01230 and VCG 01221. 

### 3.9. Presence of SIX Gene Homologues in Foc

*SIX* gene homologues were mostly conserved within the newly described VCGs and SMVs in Clade A, and among Clade B VCGs and SMVs (Table 2). The new VCG 01227, and SMVs 1 and 3, produced unique *SIX* homologues when compared with other VCGs in Clade A. VCG 01227 had a similar suite of *SIX* gene homologs as Foc VCG 0120/15 but amplified a *SIX6* homologue and no *SIX7* and *SIX8b* homologues, such as VCG 01210. In Clade B, *SIX4* homologues were absent in SMVs 4, 12 and 16, and VCG 01237, whereas the *SIX13a* homologue was not amplified for SMV 8 and SMV 10. The *SIX9c* homologue was amplified in SMV 10 and 12, and the SIX13 (d–e) homologues in VCGs 01221 and 01230. Interestingly, no *SIX4* and *SIX6* homologues were produced for the VCG 01222 BRIP 59170 [21], whereas these were produced for the same VCG CAV 853. The *SIX* gene homologues in VCG 01226 (Clade C) and SMV 2 were similar to those in Clade B, except for the absence of the *SIX13* homologue. No *SIX* gene homologues were found in the non-pathogenic isolates included in this study.

## 4. Discussion

Vegetative compatibility has been a useful tool to study the origin, diversity and migration of *formae speciales* of *F. oxysporum* [8]. It is also a trait that has been used for the management of plant pathogens with mycoviruses [54] and atoxigenic fungal strains [55]. In this study, 14 new VCGs and 17 SMVs were described for Foc, which add to the 24 known Foc VCGs. This confirms the notion that the banana Fusarium wilt pathogen is more diverse than previously anticipated [6,17,22,56]. New VCGs can develop due to mutations in the *vic/het* loci that govern vegetative compatibility [1] that may occur randomly or through selection pressure during the coevolution of host and pathogen [5,56]. The diversity in Foc could also be attributed to past sexual reproduction [57], parasexuality [58,59] and horizontal gene transfer events [21,27].

Many previously unknown VCGs described in this study are genetically related to known VCGs. This is most noticeable in the SC4 cluster, where 12 VCGs and 6 SMVs shared a narrow genetic base. The SNP ML phylogeny illustrated a comb-shaped sub-cluster in SC4 that includes VCGs in the VCG 0124/5/8/20/22 complex, as well as SMVs 13 and 14, which suggests a fast and recent demographic expansion. The VCG 0124/5/8/20/22 complex includes some of the most widely distributed Foc strains in world [6,17,20,31], and clustered basal to several distinct polytomic sub-lineages in SC4, which consist of VCG 01212 and several newly described VCGs. These sub-lineages suggest that SC4 is still an expanding lineage. It could be hypothesised that genetic drift, due to an association with a new banana host or new geographical location, might have contributed to the development of the new VCGs. An example is VCG 01212 that clustered as one of the polytomic sub-lineages within SC4. VCG 01212 is unique to East and Central Africa, but closely related to VCGs 0124/5/8/20/22 that are widespread in the Indian subcontinent, from where they were most likely introduced into Africa [60]. This hypothesis should be further investigated in a comprehensive population genomic study involving more representative isolates of each VCG from different geographical locations.

In the current study the SC3 cluster, which includes the novel VCG 01226 from Me-xico, is phylogenetically distinct from Foc Clade A and B strains. This may indicate a potentially independent evolutionary event where pathogenicity to banana developed. The clustering of VCG 01226 in Clade C should, however, in future be confirmed by including more *formae speciales* of *F. oxysporum* and a more distant outgroup for a phylogenomic analysis. A recent study on Foc samples isolated from diseased Manzano banana (Silk subgroup, AAB) in Mexico described a Foc strain (M104) with similar topology to VCG 01226 in a multi-gene phylogeny, although no VCG testing was performed [61]. While it is not anticipated that newly described VCGs would affect banana varieties differently than those in closely related known VCGs, distantly related VCGs such as VCG 01226 might have a very different host range than the known VCGs. Silk bananas are often affected by several VCGs [6] in countries that grow the variety for livelihoods and local markets. If, however, infested fields are used for large-scale monoculture production of a susceptible variety in future, this VCG might become damaging.

Non-pathogenic endophytic *F. oxysporum* isolates included in our analysis clustered with pathogenic isolates in clusters SC3, SC5 and SC7. This could indicate that VCGs and SMVs in these clusters developed from endophytic *F. oxysporum* populations. Most of the studies determining the diversity of plant pathogenic populations only focus on virulent strains causing economically important epidemics such as that of Foc tropical race 4. As a result, our understanding of genetic diversity that exists in natural populations of fungi is often distorted [62]. As non-pathogenic *F. oxysporum* strains are ubiquitous in banana host tissue and agricultural soils, the inclusion of such isolates in population studies could broaden the view of the population structure and evolutionary history [63]. The inclusion of non-pathogens in the development of molecular detection tools is also important to ensure their reliability and to avoid false positives. In this regard, Magdama et al. [64] demonstrated that the use of the intergenic spacer (IGS) ribosomal DNA locus to design Foc TR4-specific assays can be deceptive due to the high homology in the IGS between endophytic and Foc strains. Incidentally, the non-pathogenic *F. oxysporum* endophytes from Australia that were amplified with Foc TR4-specific primers developed by Dita et al. [32] did not cluster with Foc TR4 VCGs in either the multi-gene or SNP phylogenies.

Several of the new VCGs identified in this study were already widespread and could present a risk if disseminated to new regions. VCGs 01227 and 01228 seem to be of particular importance for the banana cultivar Latundan in the Philippines, as they have been present over a long temporal range. These were first recorded by Bentley et al. [17] in the Philippines, and 18 years later in Luzon and Mindanao islands by Solpot et al. [30]. VCG 01233 seems to be important in Oman on the Poovan cultivar, but also occurs in Sri-Lanka and India, possibly spread from there with movement of planting material. This, again, indicates the value of VCG analysis in understanding the migration of Foc from one country to another. The historical ties between the three countries related to trade and culture could have facilitated movement of planting material that might have spread this Foc VCG. These findings warrant further investigation into the incidence and virulence of the newly described VCGs under prevailing environmental conditions and local cultivars.

The conservation of *SIX* gene homologues within phylogenetic lineages, and their congruence with core genome phylogenies, indicate that the *SIX* homologues were vertically inherited from a common ancestor, as previously reported by Czislowski et al. [21]. For example, the newly described VCGs in SC4 had an identical suite of *SIX* gene homologues as the known VCGs 0124/8/12/20/22. SMV 1, SMV 3 and VCG 01226, however, showed a unique set of *SIX* gene homologues, which might help to predict the host range or virulence of an isolate [65,66,67]. The role of *SIX* gene homologues in the virulence of Foc to banana is, however, not clear, as only the involvement of *SIX1* and *SIX8* gene homologues has so far been experimentally demonstrated [68].

Reticulate branching discordances were observed in the SNP phylogenies that may be signatures of ongoing or past recombination events. Statistical evidence for recombination, however, was only obtained for SC6 when using the PHI test, while the other clusters were clonal. Mating type diagnosis has been determined for the known VCGs 0123 and 01217 in this cluster [20], with only the *MAT-1* present. Comparative studies conducted on the mitochondrial genome of 61 FOSC members indicated that recombination is possible between members of the same clade, but there is genetic isolation between the different clades [69]. It would be interesting to determine the mating types of new and known VCGs present in each of the clusters in the future, to indicate whether cryptic sexual reproduction is possible within each cluster. Sympatric populations will be necessary to investigate the occurrence of recombination in Foc.

It is evident that the inconsistent designation of Foc lineages, when using different techniques, can cause confusion and this should be resolved. However, the authors of this study do not agree with the renaming of Foc lineages to separate *Fusarium* species [22] without carefully considering the scientific and practical merit thereof and potential confusion created for practitioners and legislators [70,71]. While the subdivision of the FOSC into species is inevitable, the authors of this study believe that this action requires a more pragmatic approach, with consensus and support of peers in the scientific community.

## 5. Conclusions

This study updated the genetic structure of the banana Fusarium wilt pathogen. It revealed that Foc is divided into eight well-defined clusters that correlated well with the clades and lineages described previously [15,17,18,19,20,22,28]. Several new VCGs and SMVs were described in addition to the 24 known Foc VCGs. This study clearly demonstrated the contrasts in diversity between Foc Clades A and B, with the Clade A cluster including the closely related Foc TR4 and STR4 isolates, while Clade B include Foc race 1 and 2 isolates in five clusters with a high divergence between SC4 and SC5–8. Understanding the diversity of Foc is important to evaluate banana varieties for resistance to Fusarium wilt. It is, therefore, important that the newly described VCGs be included in resistance screening of banana improvement programmes. It is also important to monitor the distribution, spread and impact of SMVs to prevent the development of new epidemics, as was the case for VCG 01213/16 when Cavendish bananas were introduced on commercial plantations in Indonesia and Malaysia.

## Figures and Tables

**Table 1:** Summary of phylogenetic clades and Snapclust grouping among different *Fusarium oxysporum* f. sp. *cubense* vegetative compatibility groups.

Clade	SC Grouping	VCGs
A	1	0120/15, 0122, 0126, 0129/11, 01210, 01219, 01227, SMV1, SMV2
	2	0121, 01213/16, SMV3
C	3	BRIP62834, 01226
B	4	0124/5, 0128, 01212, 01220, 01222, 01228, 01229, 01232,01233, 01236, 01237, SMV4–7, SMV13, SMV 17
	5	01214, 01231, SMV12, BRIP62833
	6	0123, 01217, 01223, 01224, 01225, 01238, SMV 15–16
	7	01218, 01234, 01235, SMV8–11, BRIP62618, BRIP62577
	8	01221, 01230

**Table 2:** *Secreted in xylem* gene distribution within different *Fusarium oxysporum* f. sp. *cubense* vegetative compatibility groups.

		*SIX* Gene															
Culture Collection	VCG	1a–g	1h	1i	2	4	6	7	8a	8b	9a	9b	9c	10	13a	13b + c	13d + e
44,012 *	0120	+	-	-	+	+	-	+	+	+	+	-	-	-	-	-	-
62,892 *	0122	+	-	+	-	-	-	-	+	-	+	-	-	-	-	+	-
59,161 *	0126	+	-	-	+	+	-	+	+	+	+	-	-	-	-	-	-
40,255 *	0129	+	-	-	+	+	-	+	+	+	+	-	-	-	-	-	-
26,029 *	01210	+	-	-	+	+	+	-	-	-	+	-	-	-	+	-	-
39,259 *	01211	+	-	-	+	+	-	+	+	+	+	-	-	-	-	-	-
36,112 *	01215	+	-	-	+	+	-	+	+	+	+	-	-	-	-	-	-
63,261 *	01219	+	-	-	+	+	-	+	+	+	+	-	-	-	-	-	-
615	01227	+	-	-	+	+	+	-	+	-	+	-	-	-	-	-	-
59,028 *	0120/15	+	-	-	+	+	-	+	+	+	+	-	-	-	-	-	-
600	SMV 1	+	-	-	-	+	+	+	+	+	+	-	-	-	-	-	-
818	SMV 2	+	-	-	-	+	+	-	-	-	+	-	-	-	-	-	-
62,962 *	0121	+	+	+	+	+	+	+	+	-	+	-	-	+	-	-	+
40,340 *	01213	+	+	+	+	+	+	-	+	-	+	-	-	-	+	-	+
59,049 *	01216	+	+	+	+	+	+	-	+	-	+	-	-	-	-	-	+
1683	01213/16	+	+	+	+	+	+	-	+	-	+	-	-	-	+	-	+
2357	01213/16	+	+	+	+	+	+	-	+	-	+	-	-	-	+	-	+
3372	01213/16	+	+	+	+	+	+	-	+	-	+	-	-	-	+	-	-
616	SMV 3	+	-	-	+	+	+	-	+	+	+	-	-	+	-	-	-
1024	01226	+	-	-	-	+	+	-	-	-	+	-	-	-	-	-	-
62,933 *	0124	+	-	-	-	+	+	-	-	-	+	-	-	-	+	-	-
62,957 *	0125	+	-	-	-	+	+	-	-	-	+	-	-	-	+	-	-
22,887 *	0128	+	-	-	-	+	+	-	-	-	+	-	-	-	-	-	-
62,955 *	01212	+	-	-	-	+	+	-	-	-	+	-	-	-	+	-	-
58,803 *	01220	+	-	-	-	+	+	-	-	-	+	-	-	-	+	-	-
853	01222	+	-	-	-	+	+	-	-	-	+	-	-	-	+	-	-
59,170 *	01222	+	-	-	-	-	-	-	-	-	+	-	-	-	+	-	-
620	01228	+	-	-	-	+	+	-	-	-	+	-	-	-	+	-	-
898	01229	+	-	-	-	+	+	-	-	-	+	-	-	-	+	-	-
1907	01232	+	-	-	-	+	+	-	-	-	+	-	-	-	+	-	-
1910	01232	+	-	-	-	+	+	-	-	-	+	-	-	-	+	-	-
1942	01233	+	-	-	-	+	+	-	-	-	+	-	-	-	+	-	-
1960	01233	+	-	-	-	+	+	-	-	-	+	-	-	-	+	-	-
1970	01233	+	-	-	-	+	+	-	-	-	+	-	-	-	+	-	-
2413	01236	+	-	-	-	+	+	-	-	-	+	-	-	-	+	+	-
2407	01236	+	-	-	-	+	+	-	-	-	+	-	-	-	+	+	-
3142	01237	+	-	-	-	-	+	-	-	-	+	-	-	-	+	-	-
3143	01237	+	-	-	-	-	+	-	-	-	+	-	-	-	+	-	-
58,813 *	0124/22	+	-	-	-	+	+	-	-	-	+	-	-	-	+	-	-
3475	0124/5	+	-	-	-	+	+	-	-	-	+	-	-	-	+	-	-
3478	0124/5	+	-	-	-	+	+	-	-	-	+	-	-	-	+	-	-
3481	0124/5	+	-	-	-	+	+	-	-	-	+	-	-	-	+	-	-
1004	0128/20	+	-	-	-	+	+	-	-	-	+	-	-	-	+	-	-
3474	SMV 13	+	-	-	-	+	+	-	-	-	+	-	-	-	+	-	-
3484	SMV 14	+	-	-	-	+	+	-	-	-	+	-	-	-	+	-	-
3127	SMV 17	+	-	-	-	+	+	-	-	-	+	-	-	-	+	-	-
624	SMV 4	+	-	-	-	-	+	-	-	-	+	-	-	-	+	-	-
1927	SMV 5	+	-	-	-	+	+	-	-	-	+	-	-	-	+	-	-
1933	SMV 6	+	-	-	-	+	+	-	-	-	+	-	-	-	+	-	-
1974	SMV 7	+	-	-	-	+	+	-	-	-	+	-	-	-	+	-	-
25,609 *	01214	+	-	-	-	-	-	-	-	-	+	-	+	-	+	-	-
1833	01231	+	-	-	-	+	+	-	-	-	+	-	-	-	+	-	-
3128	01231	+	-	-	-	+	+	-	-	-	+	-	-	-	+	-	-
3130	01231	+	-	-	-	+	+	-	-	-	+	-	-	-	+	-	-
2611	Non-pathogen	-	-	-	-	-	-	-	-	-	-	-	-	-	-	-	-
2633	Non-pathogen	-	-	-	-	-	-	-	-	-	-	-	-	-	-	-	-
2734	SMV 12	+	-	-	-	-	+	-	-	-	+	-	+	-	+	-	-
62,895 *	0123	+	-	-	-	+	+	-	-	-	+	-	-	-	+	-	-
58,698 *	01217	+	-	-	-	+	+	-	-	-	+	-	-	-	+	-	-
36,116 *	01223	+	-	-	-	+	+	-	-	-	+	-	-	-	+	-	-
909	01225	+	-	-	-	+	+	-	-	-	+	-	-	-	+	-	-
3351	01238	+	-	-	-	+	+	-	-	-	+	-	-	-	+	-	-
3371	01238	+	-	-	-	+	+	-	-	-	+	-	-	-	+	-	-
3534	SMV 15	+	-	-	-	+	+	-	-	-	+	-	-	-	+	-	-
3540	SMV 16	+	-	-	-	-	+	-	-	-	+	-	-	-	+	-	-
63,259 *	01218	+	-	-	-	+	+	-	-	-	+	+	-	-	+	-	-
2266	01234	+	-	-	-	+	+	-	-	-	+	-	-	-	+	-	-
2267	01234	+	-	-	-	+	+	-	-	-	+	-	-	-	+	-	-
2271	SMV 10	+	-	-	-	+	+	-	-	-	+	-	+	-	-	-	-
2351	SMV 11	+	-	-	-	+	+	-	-	-	+	+	-	-	+	-	-
2075	SMV 8	+	-	-	-	+	+	-	-	-	+	-	-	-	-	-	-
2257	SMV 9	+	-	-	-	+	+	-	-	-	+	-	-	-	+	-	-
2281	01235	+	-	-	-	+	+	-	-	-	+	-	-	-	+	-	-
2287	01235	+	-	-	-	+	+	-	-	-	+	-	-	-	+	-	-
1003	01221	+	-	-	-	-	-	-	-	-	+	-	-	-	+	-	+
36,118 *	01221	+	-	-	-	-	-	-	-	-	+	-	-	-	+	-	+
1011	01230	+	-	-	-	-	+	-	-	-	+	-	-	-	+	-	+

* Presence and absence of *SIX* gene homologues information included from the study of Czislowski et al. (2018) [12].

## Data Availability

The data that support the findings of this study not included in the paper or in supplementary files are available from the corresponding author upon reasonable request.

## Supplementary Materials

The following are available online at https://www.mdpi.com/article/10.3390/microorganisms10020269/s1, Figure S1. Frequency distributions of call rate for (A) SilicoDArT loci and (B) SNPs; Figure S2: Correlation between DArTseq and DArTseq SNPs; Figure S3. Determination of the optimal number of clusters, using the Akaike Information Criterion; Figure S4. NeighborNet networks of the SC clusters; Table S1. Summary of Foc isolates phenotyped and genotyped; Table S2. PHI test of each SC cluster.
