# Supplementary material for: A Polyphasic Approach Reveals Novel Genotypes and Updates the Genetic Structure of the Banana Fusarium Wilt Pathogen
Source: Microorganisms. 2022 Jan 25;10(2):269. doi: 10.3390/microorganisms10020269 (PMC8876670; doi:10.3390/microorganisms10020269)
Supplement: Supplementary file 1 [file microorganisms-10-00269-s001.zip › Figure S2.pdf]

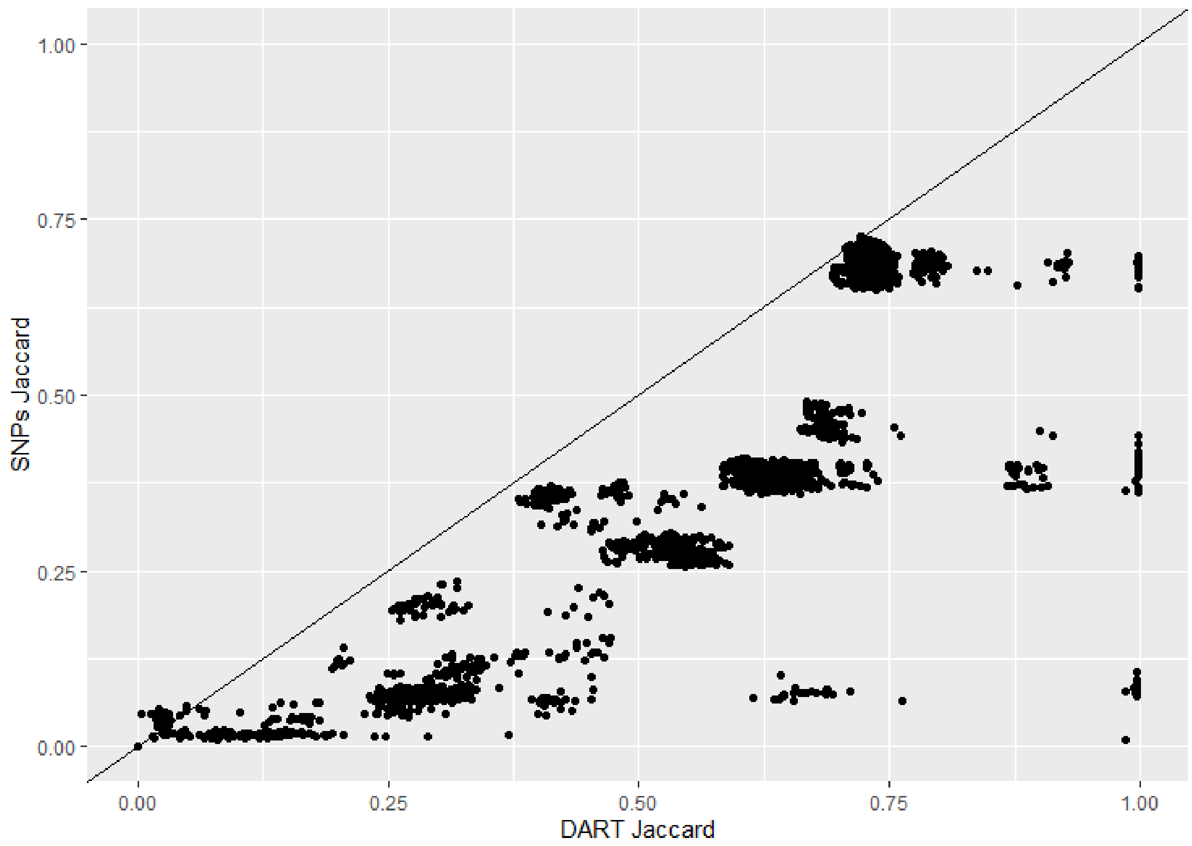

Figure S2: Correlation between pairwise Jaccard distances between each genotype pair (n=84), based on DArTseq loci and DArTseq SNPs. The congruence between both distance matrices were assessed using the Mantel correlation test. The Mantel correlation coefficient was 0.823 indicating highly significant correlation (P= 0.0001).
