# Supplementary material for: A Polyphasic Approach Reveals Novel Genotypes and Updates the Genetic Structure of the Banana Fusarium Wilt Pathogen
Source: Microorganisms. 2022 Jan 25;10(2):269. doi: 10.3390/microorganisms10020269 (PMC8876670; doi:10.3390/microorganisms10020269)
Supplement: Supplementary file 1 [file microorganisms-10-00269-s001.zip › Figure S3.pdf]

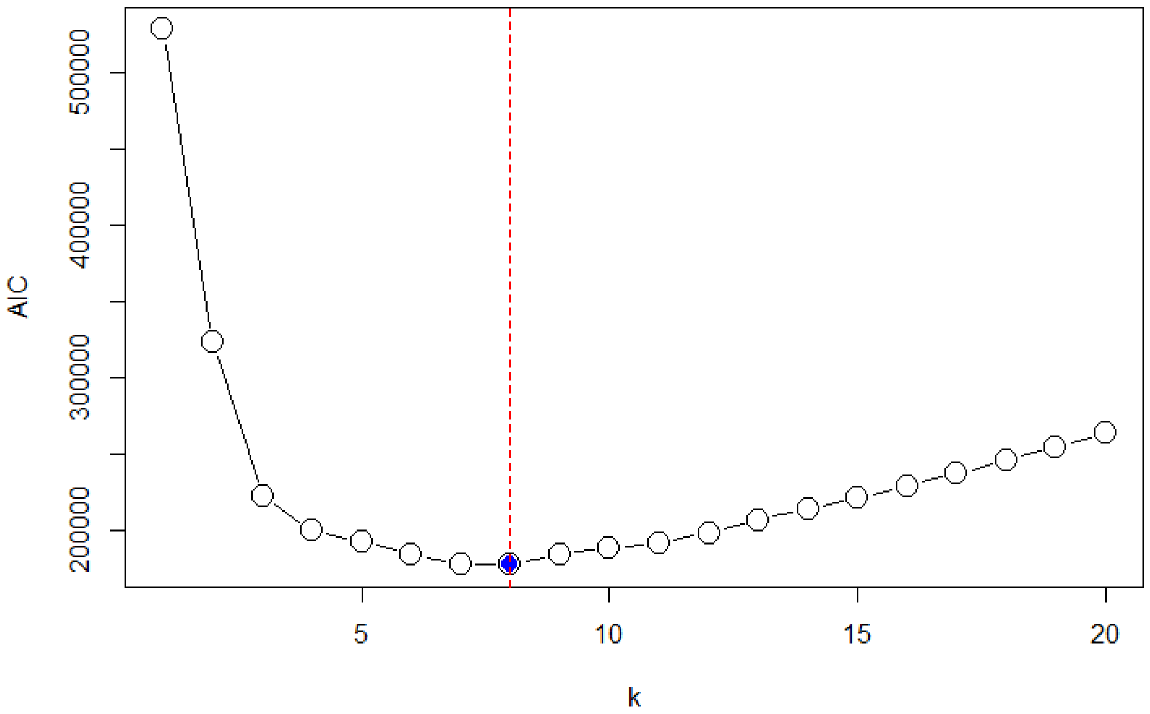

Figure S3. Determination of the optimal number of clusters, using the Akaike Information Criterion (AIC).
