# Supplementary material for: A Polyphasic Approach Reveals Novel Genotypes and Updates the Genetic Structure of the Banana Fusarium Wilt Pathogen
Source: Microorganisms. 2022 Jan 25;10(2):269. doi: 10.3390/microorganisms10020269 (PMC8876670; doi:10.3390/microorganisms10020269)
Supplement: Supplementary file 1 [file microorganisms-10-00269-s001.zip › Figure S4.pdf]

A. SC-01

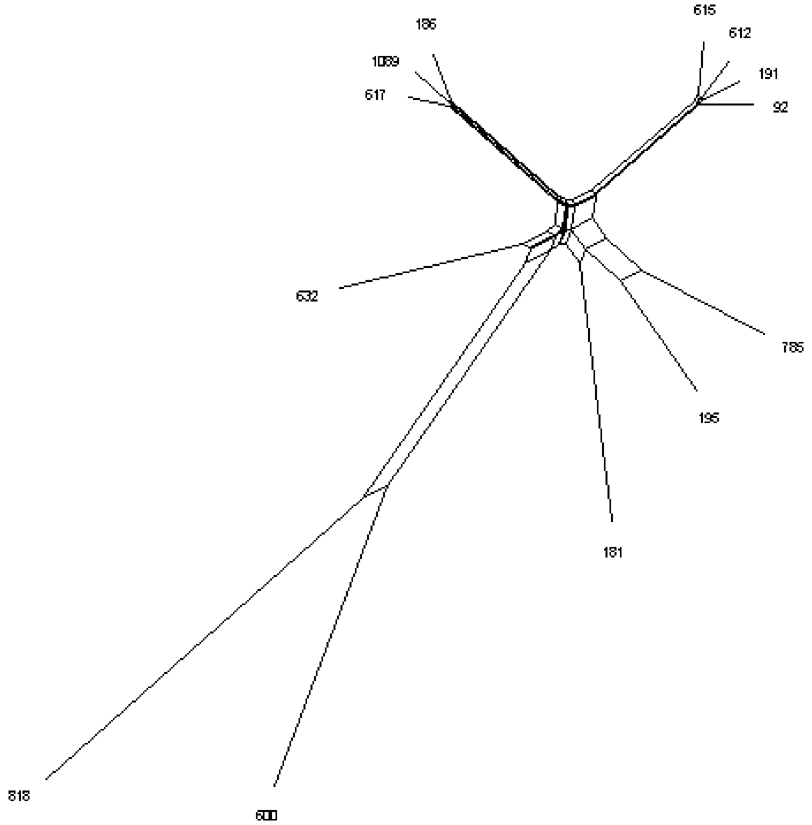
186
1089
617
615
612
191
92
632
785
195
181
818
600

B. SC-02

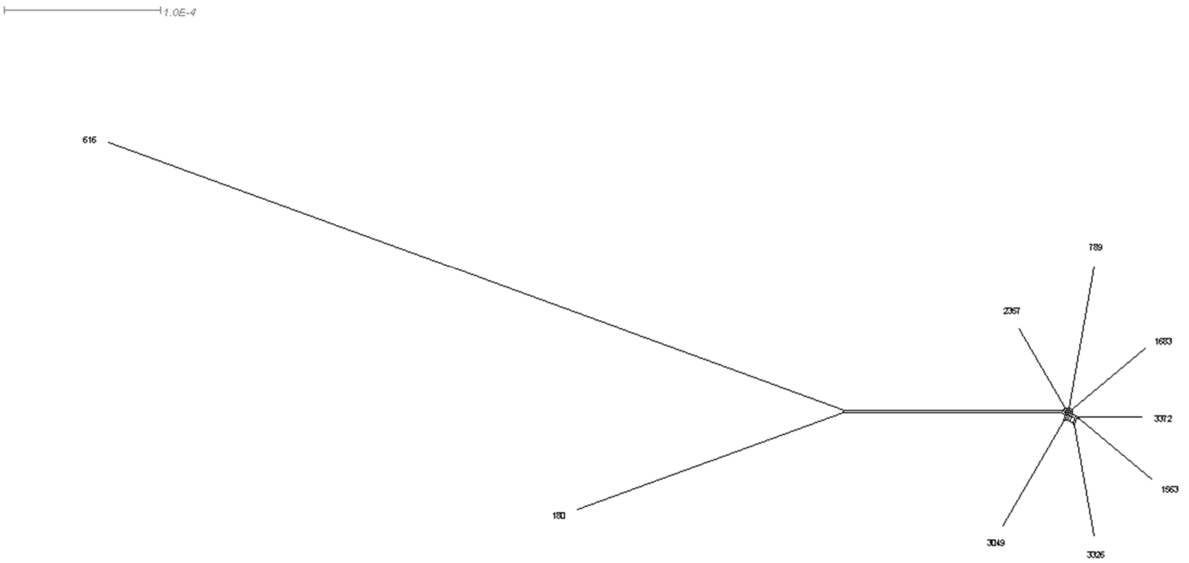
1.0E-4
616
789
2367
1683
3372
1563
180
3049
3326

C. SC-01 and 02

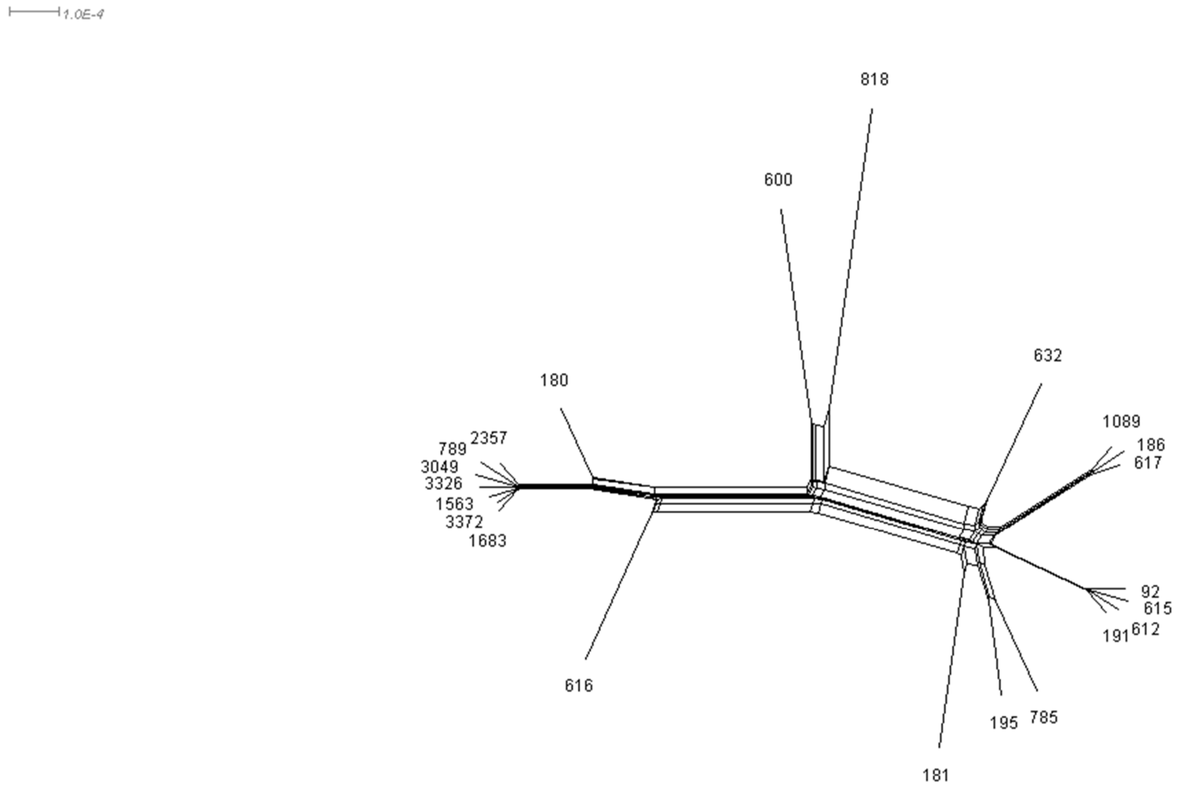

D. SC-04

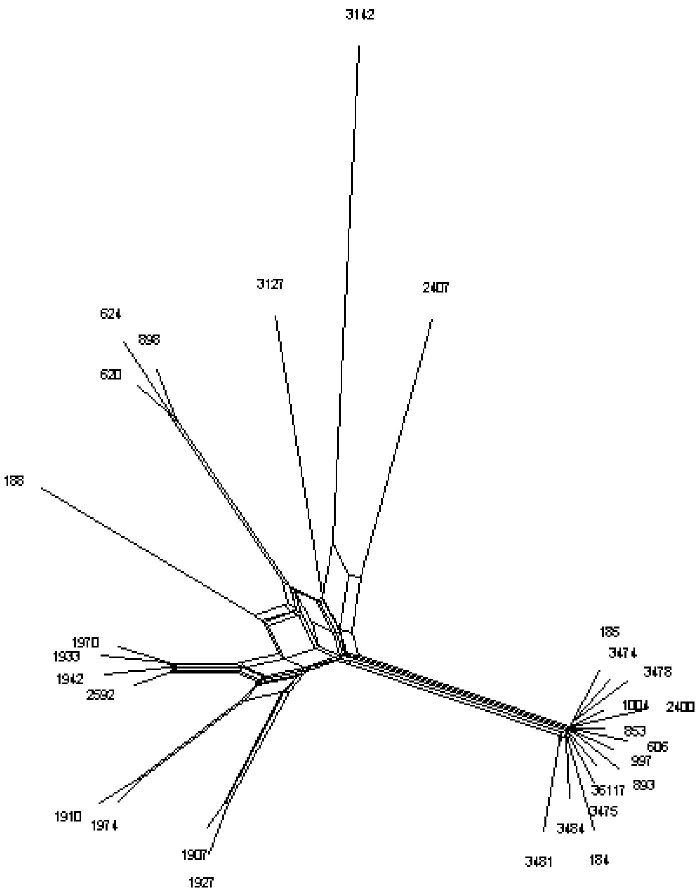

E. SC-05

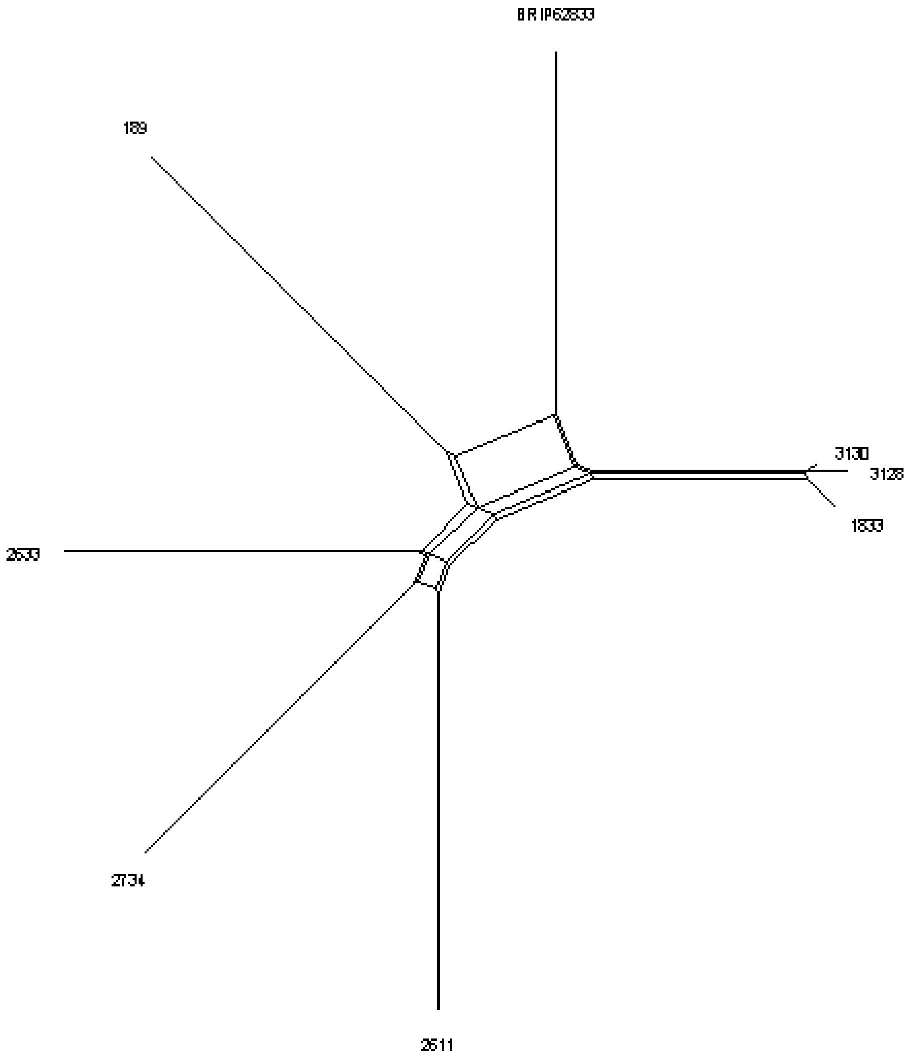

F. SC-06

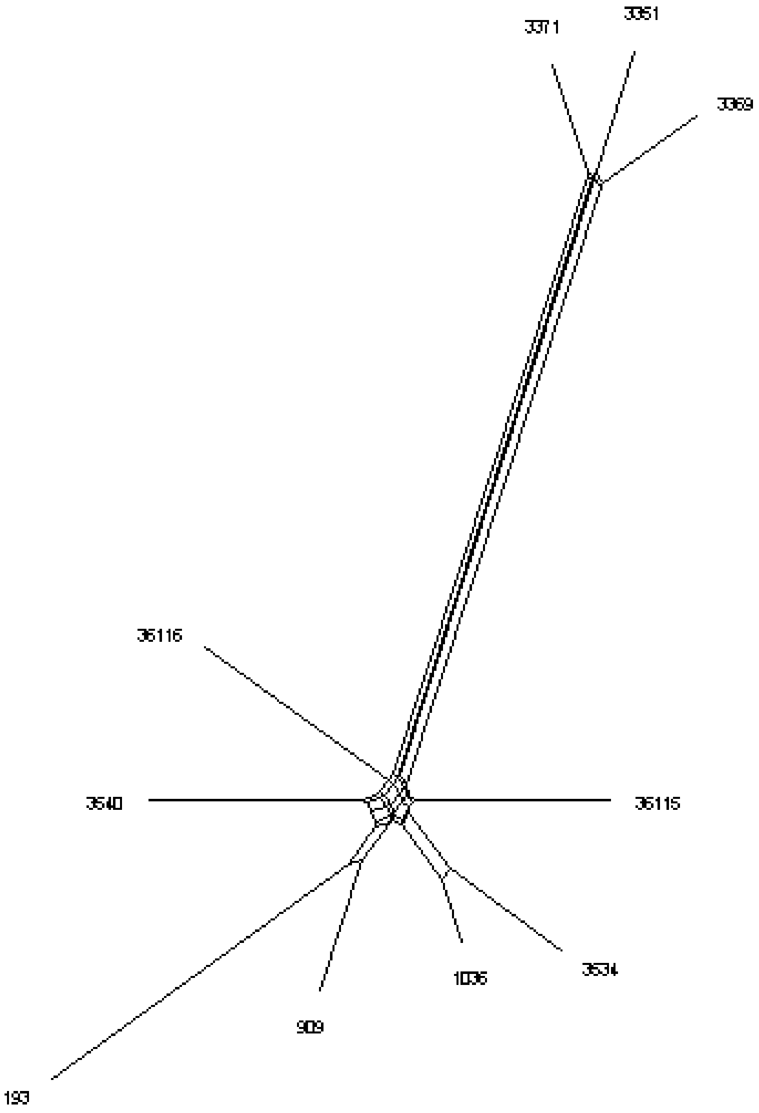

G. SC-07

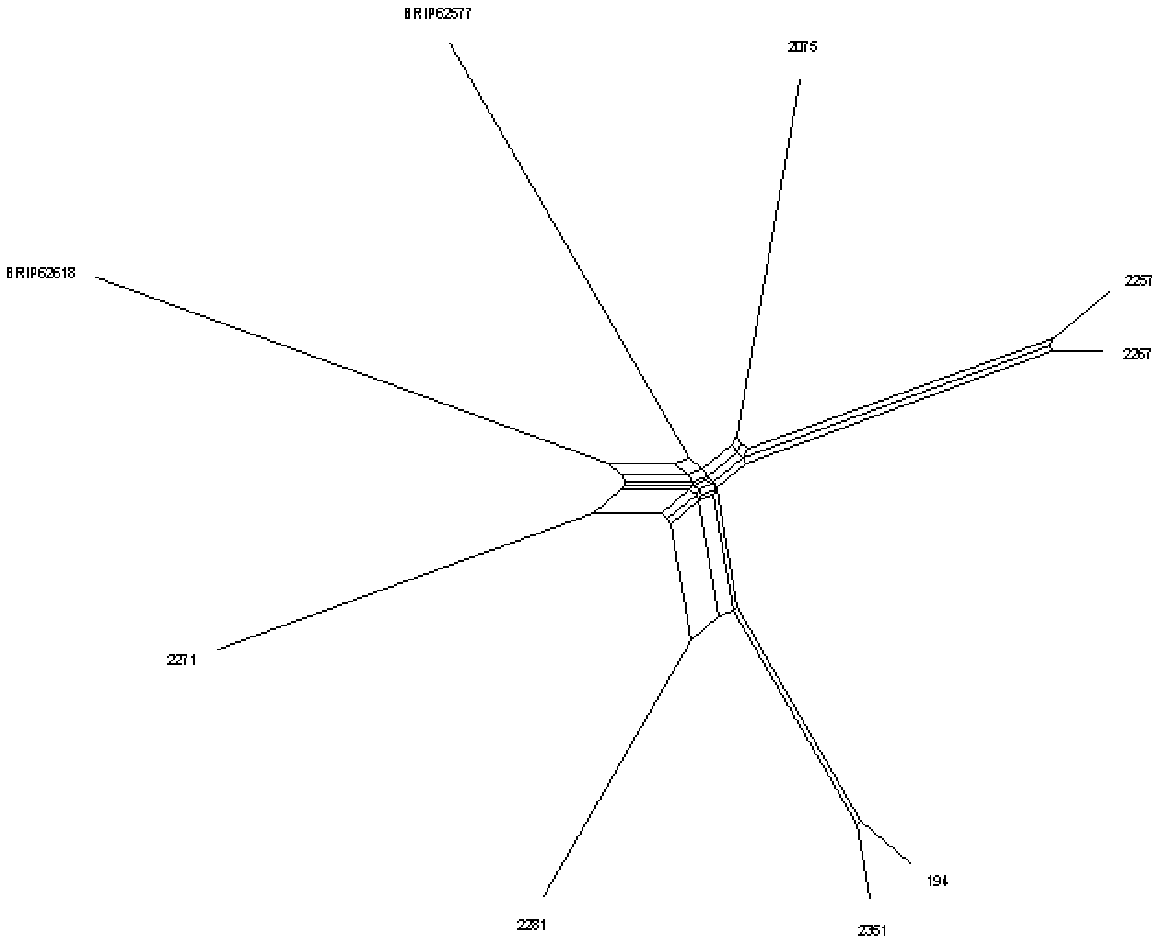

Figure S4. NeighborNet networks of the Snapclust-DAPC clusters (SC clusters) using SplitsTree 4.
