# Supplementary material for: A Polyphasic Approach Reveals Novel Genotypes and Updates the Genetic Structure of the Banana Fusarium Wilt Pathogen
Source: Microorganisms. 2022 Jan 25;10(2):269. doi: 10.3390/microorganisms10020269 (PMC8876670; doi:10.3390/microorganisms10020269)
Supplement: Supplementary file 1 [file microorganisms-10-00269-s001.zip › Table S2.pdf]

Table S2. Results of Pairwise homoplasy Index (PHI) test on each SC cluster within the Foc dataset, using SplitsTree 4.

PHI test	SC01	SC02	SC04	SC05	SC06	SC07
Mean	0.32	0.323	0.393	0.389	0.173	0.377
Variance	0	0.001	0.001	0.001	0.001	0.001
Observed	0.304	0.286	0.398	0.362	0.129	0.387
P-value	0.204	0.169	0.592	0.173	0.023	0.652
Interpretation	clonal	clonal	clonal	clonal	recombinant	clonal
