# Supplementary figures and images for: A Polyphasic Approach Reveals Novel Genotypes and Updates the Genetic Structure of the Banana Fusarium Wilt Pathogen
Source: Microorganisms. 2022 Jan 25;10(2):269. doi: 10.3390/microorganisms10020269 (PMC8876670; doi:10.3390/microorganisms10020269)

A

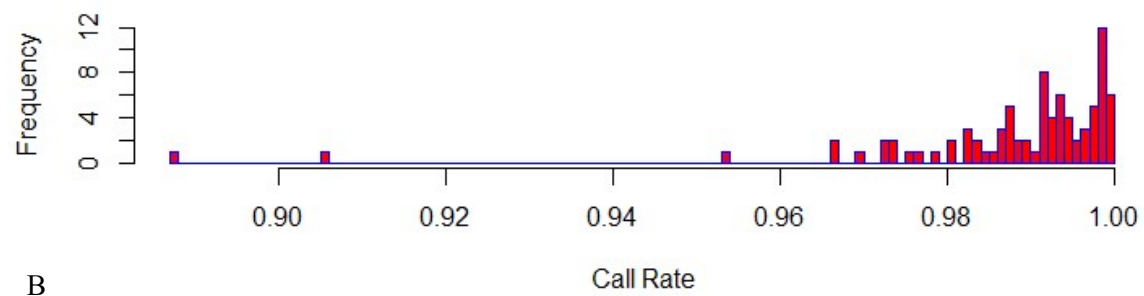

B

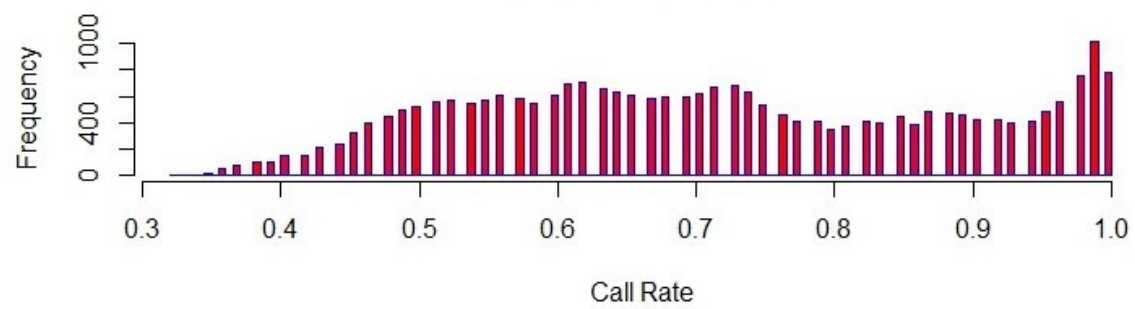

**Figure S1.** Frequency distributions of call rate for (A) SilicoDArT loci and (B) SNPs.

Supplement: Supplementary file 1 [file microorganisms-10-00269-s001.zip › Figure S1.pdf]
